# The efficacy and safety of induction chemotherapy combined with concurrent chemoradiotherapy versus concurrent chemoradiotherapy alone in nasopharyngeal carcinoma patients: a systematic review and meta-analysis

**DOI:** 10.1186/s12885-020-06912-3

**Published:** 2020-05-06

**Authors:** Bi-Cheng Wang, Bo-Ya Xiao, Guo-He Lin, Chang Wang, Quentin Liu

**Affiliations:** 1 Cancer Center, Union Hospital, Tongji Medical College, Huazhong University of Science and Technology, Wuhan, 430022 China; 2 Eastern Hepatobiliary Surgery Hospital, Second Military Medical University, Shanghai, 200438 People’s Republic of China; 3 Department of Oncology, the Second Affiliated Hospital of Anhui Medical University, Hefei, 230601 China; 4 Institute of Anatomy, University of Bern, CH-3012 Bern, Switzerland; 5 State Key Laboratory of Oncology in South China, Collaborative Innovation Center for Cancer Medicine, Cancer Center, Sun Yat-sen University, Guangzhou, 510060 China

**Keywords:** Induction chemotherapy, Concurrent chemoradiotherapy, Survival, Nasopharyngeal carcinoma, Meta-analysis

## Abstract

**Background:**

Induction chemotherapy (IC) combined with concurrent chemoradiotherapy (CCRT) has been recommended as the first-line therapy for locoregional nasopharyngeal carcinoma (NPC). Due to the different chemotherapeutic drugs used in the IC and CCRT, the results remain controversial.

**Methods:**

PubMed, EMBASE, Web of Science, and Cochrane Library databases were systematically retrieved to search potentially eligible clinical trials up to Sep 11, 2019. Eligible studies were registered and prospective randomized controlled clinical trials.

**Results:**

From 526 records, nine articles including seven randomized controlled clinical trials were eligible, with a total of 2311 locoregional advanced NPC patients. IC + CCRT had significantly lower risks of death (3-year hazard ratio [HR]: 0.70, 95% confidence interval [CI] 0.55–0.89, *p* = 0.003; 5-year HR: 0.77, 95% CI 0.62–0.94, *p* = 0.01), disease progression (3-year HR: 0.67, 95% CI 0.55–0.80, *p* < 0.001; 5-year HR: 0.70, 95% CI 0.58–0.83, *p* < 0.0001), distant metastasis (3-year HR: 0.58, 95% CI 0.45–0.74, *p* < 0.0001; 5-year HR: 0.69, 95% CI 0.55–0.87, *p* = 0.001) and locoregional relapse (3-year HR: 0.69, 95% CI 0.50–0.95, *p* = 0.02; 5-year HR: 0.66, 95% CI 0.51–0.86, *p* = 0.002) than CCRT. Compared with CCRT, IC + CCRT showed higher relative risks of grade 3 or more neutropenia, thrombocytopenia, nausea, vomiting and hepatotoxicity throughout the course of treatment, and higher relative risks of grade ≥ 3 thrombocytopenia and vomiting during CCRT.

**Conclusion:**

IC combined with CCRT significantly improved the survival in locoregional advanced NPC patients. Moreover, toxicities were well tolerated during IC and CCRT. Further clinical trials are warranted to confirm the optimal induction chemotherapeutic regimen in the future.

## Highlights

1. IC combined with CCRT significantly improved the survival outcomes of patients with locoregional advanced NPC.
2. IC combined with CCRT showed higher relative risks of grade 3 or more neutropenia, thrombocytopenia, nausea, vomiting and hepatotoxicity throughout the course of treatment, and higher relative risks of grade 3 or more thrombocytopenia and vomiting during CCRT.

## Background

Nasopharyngeal carcinoma (NPC) is one of head and neck tumors with an unbalanced endemic distribution and a high prevalence in Southeast Asia, Southeast China, and North Africa [1]. More than two decades ago, locoregionally advanced NPC had an unfavorable prognosis. Since the administration of concurrent chemoradiotherapy (CCRT), the survival outcomes have been significantly improved [2, 3].

However, there are still over 20% of patients with locoregionally advanced NPC living for less than 5 years [3]. In the European Society for Medical Oncology (ESMO) clinical practice guideline, CCRT is suggested to treat locoregionally advanced NPC (category 1A), while induction chemotherapy (IC) combined with CCRT is recommended to stage IV NPC patients (category 2B) [4]. Nevertheless, this guideline has not been updated since 2012.

In the National Comprehensive Cancer Network (NCCN) clinical practice guideline for patients with locoregionally advanced NPC, the preferred recommendation is participating in clinical trials, while the category 2A and 2B recommendations are, respectively, IC followed by CCRT and CCRT alone [5].

In the past decade, considerable studies on IC for NPC have been carried out. Among these clinical trials, different chemotherapeutic drugs and different doses or cycles of the IC were administered. However, owing to multiple clinical trials showing different results, adding IC to CCRT remains controversial.

Accordingly, in this systematic review and meta-analysis, we compared the IC plus CCRT with CCRT alone in NPC patients to analyze the 3-year/5-year survival outcomes and grade ≥ 3 toxicities in the registered and prospective clinical studies.

## Methods

This analysis was conducted according to the Preferred Reporting Items for Systematic Reviews and Meta-analyses guideline (PRISMA) [6].

### Search strategy

A systematic literature search was performed in PubMed, EMBASE, Web of Science, and Cochrane Library databases to identify all relevant records up to Sep 11, 2019. Search terms included: “induction chemotherapy”, “concurrent chemoradiotherapy”, “nasopharyngeal carcinoma”, and “randomized controlled trial or randomized clinical trial or clinical trial or trial”. The references of relevant articles were manually searched for more clinical studies. The search records were uploaded into EndNote software (http://endnote.com/) for further review.

### Selection criteria

All of the eligible clinical trials should meet the following inclusion criteria: (1) prospective studies in previously untreated patients with NPC, (2) all eligible studies were registered clinical trials and provided the registered numbers, (3) only randomized controlled clinical studies were eligible, (4) in randomized controlled studies, the experiment group was treated with IC combined with CCRT, and the control group was treated with CCRT alone, (5) neoadjuvant chemotherapy described in the articles was deemed as induction chemotherapy, (6) IC or CCRT combined with target therapy was excluded, (7) because of the absence of complete efficacy and safety data, conference abstracts were excluded, (8) studies were published in English. Any disagreements were resolved by discussion.

### Data extraction and quality assessment

The primary outcome was overall survival (OS), failure-free survival (FFS), distant metastasis-free survival (DMFS) and locoregional relapse-free survival (LRFS), and the second outcome was toxicity. FFS was defined as the date of randomization to documented disease progression (the date of locoregional/distant failure or death from any cause, whichever occurred first). Two authors (BW and BX) independently extracted information from the full texts and supplementary materials. Any discrepancies were resolved by consensus. The following details were extracted from each eligible clinical trial: first author, publication year, inclusion period, registered number, study design, number of patients, mean age, median follow-up, therapeutic regimens, OS, FFS, DMFS, LRFS, survival rate, and adverse events. The Jadad scoring scale was used to evaluate the methodological quality of each eligible trial by two authors (BW and BX) [7].

### Statistical analysis

Survival outcomes (OS, FFS, DMFS and LRFS) from randomized controlled studies were assessed by hazard ratio (HR) with 95% confidence interval (CI) using Cochrane Collaboration’s Information Management System (RevMan) software (version 5.3). Toxicities were calculated as risk ratios (RRs) and 95% CIs. The chi-squared (χ^2^) tests and *I*^2^ statistic percentages were used to test and quantify the heterogeneity. A fixed-effects model (Mantel-Haenszel method) was adopted in the calculations if *I*^2^ ≤ 50%, otherwise, a random-effect model was applied. When *p* < 0.05, the differences were considered statistically significant.

## Results

### Eligible studies and characteristics

**Fig. 1:**
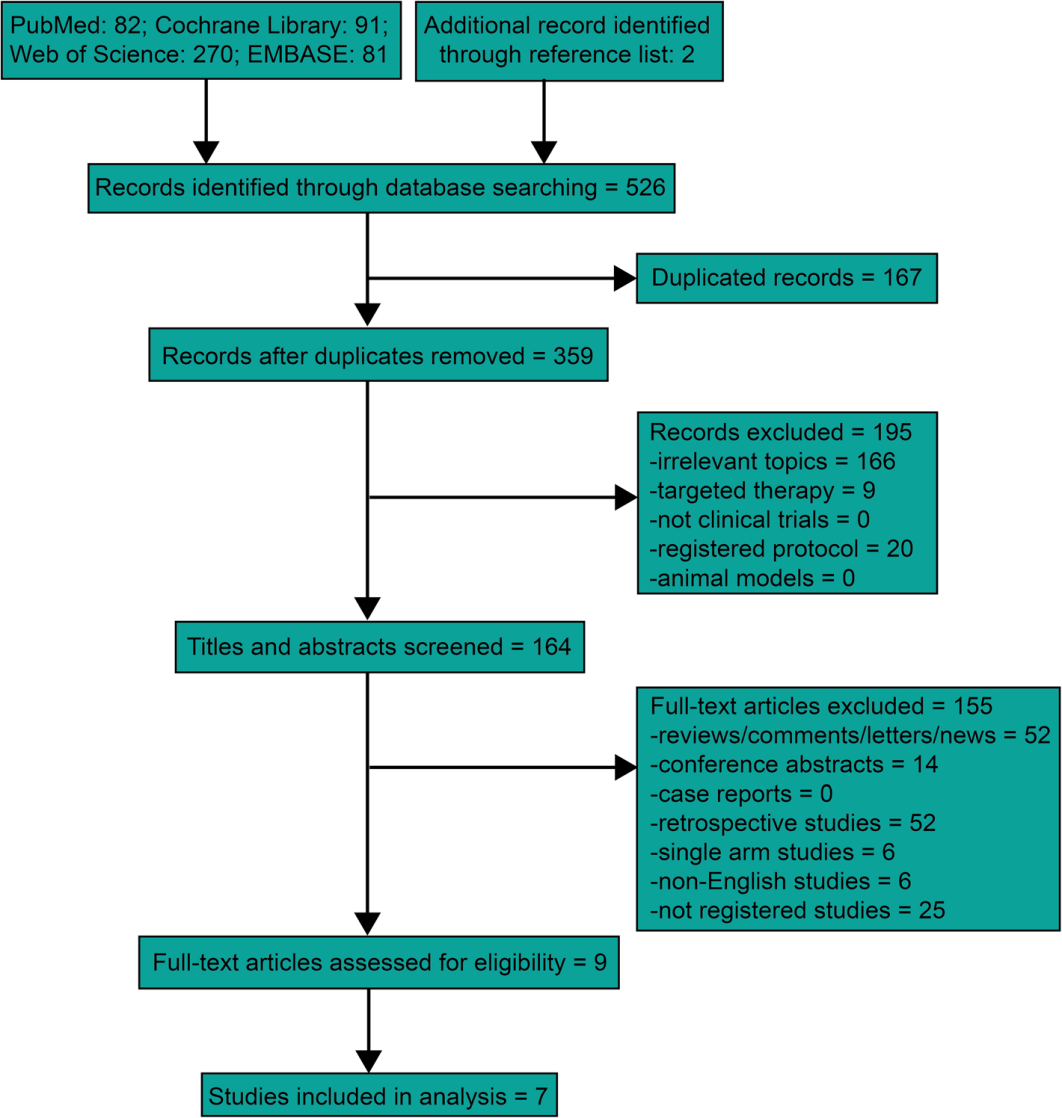
Flow chart of the selection process

Our search of the PubMed, EMBASE, Web of Science, and Cochrane Library databases identified 524 relevant publications. Two additional records were identified through reference lists. 167 duplicated records were removed. After screening the titles and abstracts, 195 records were excluded. After eligibility assessment, a total of nine studies were selected for inclusion in the systematic review, comprising seven randomized controlled studies (Fig. 1) [8–16]. Table 1 showed the basic characteristics of the eligible clinical trials. Table 2 displayed the details of therapeutic regimens and rates of OS, FFS, objective response, and grade ≥ 3 toxicities in the selected studies.

**Table 1:** Characteristics of the eligible studies

Study	Year	Inclusion period	Register	Region/ Country	Phase	Group	No.patients	No.male	Mean Age (range, year)	Stage	AJCC	Median follow-up (years)	Jadad score
Fountzilas [8]	2012	2003–2008	ACTRN 12609000730202	Europe	II	IC + CCRT CCRT	72 69	55 48	49 (19–82) 51 (15–79)	IIb-IVb	6th	4.6	4
Tan [9]	2015	2004–2012	CDR0000657121	Singapore	II/III	IC + CCRT CCRT	86 86	71 63	49 (42–55) 52 (44–57)	III-IVb	5th	3.4 3.2	3
Sun [10]/ Li [11]	2016/2019	2011–2013	NCT01245959	China	III	IC + CCRT CCRT	241 239	193 174	42 (36–49) 44 (39–50)	III-IVb	7th	6	3
Cao [12]/ Yang [13]	2017/2019	2008–2015	NCT00705627	China	III	IC + CCRT CCRT	238 238	173 190	44 (19–65)42 (21–66)	III-IVb	6th	6.9	3
Frikha [14]	2018	2009–2012	NCT00828386	France/ Tunisia	III	IC + CCRT CCRT	42 41	28 32	46 48	T2b, T3, T4 and/or N1-N3, M0	NR	3.6	3
Hong [15]	2018	2003–2009	NCT00201396	China	III	IC + CCRT CCRT	239 240	176 179	45 (15–69) 47 (19–70)	IVa-IVb	5th	6	3
Zhang [16]	2019	2013–2016	NCT01872962	China	III	IC + CCRT CCRT	242 238	182 164	46 (18–64) 45 (20–64)	III-IVb	7th	3.6	3

*IC* induction chemotherapy; *CCRT* concurrent chemoradiotherapy; *AJCC* American Joint Cancer Committee; *NR* not reported

**Table 2:** Therapeutic regimens, survival outcomes and toxicities in eligible studies

Study	Induction chemotherapy	Radiotherapy	Concurrent chemotherapy	IC + CCRT|CCRT					
				OS rate	FFS rate	DMFS rate	LRFS rate	ORR	Grade ≥ 3 AE rate
Fountzilas 2012 [8]	Epi 75 mg/m^2^, Pac 175 mg/m^2^ and DDP 75 mg/m^2^ every 21 days for 3 cycles	2D-CRT, 3D-CRT	DDP 40 mg/m^2^ every week	3-year: 66.6%*|*71.8%	3-year: 64.5%*|*63.5%	FE	FE	83%*|*85%	FE
Tan 2015 [9]	Gem 1000 mg/m^2^, CBP area under the concentration-time-curve 2.5, and Pac 70 mg/m^2^ (day 1 and 8) every 21 days for 3 cycles	2D-CRT, IMRT	DDP 40 mg/m^2^ every week	3-year: 94.3%*|*92.3%	3-year: 74.9%|67.4%	3-year: 83.8%|79.9%	FE	FE	52%*|*37%^b^
Sun/Li 2016/2019 [10] [11]	Doc 60 mg/m^2^, DDP 60 mg/m^2^ and 5-FU 600 mg/m^2^ every 21 days for 3 cycles	IMRT	DDP 100 mg/m^2^ every 21 days for 3 cycles	3-year: 92.1%*|*86.2%; 5-year: 85.6%*|*77.7%	3-year: 80.1%*|*72.0%; 5-year: 77.4%*|*66.4%	3-year: 88.8%*|*82.0%; 5-year: 88.0%*|*79.8%^b^	3-year: 91.7%*|*87.4%; 5-year: 90.7%*|*83.8%^b^	98.8%*|*100%	72.8%*|*53.8%
Cao/Yang 2017/2019 [12] [13]	DDP 80 mg/m^2^ and 5-FU 800 mg/m^2^ (day 1–5) every 21 days for 2 cycles	2D-CRT, IMRT	DDP 80 mg/m^2^ every 21 days for 3 cycles	3-year: 88.2%*|*88.5%; 5-year: 80.8%*|*76.8%^b^	3-year: 82.0%*|*74.1%; 5-year: 73.4%*|*63.1%^b^	3-year: 86.0%*|*82.0%; 5-year: 82.8%*|*73.1%^b^	3-year: 94.3%*|*90.8%; 5-year: 87.9%*|*85.0%	98.7%*|*99.2%	66.3%|49.1%^ab^
Frikha 2018 [14]	Doc 75 mg/m^2^, DDP 75 mg/m^2^ and 5-FU 750 mg/m^2^/day day (1–5) every 21 days for 3 cycles	IMRT, non-IMRT	DDP 40 mg/m^2^ every week	3-year: 86.3%*|*68.9%	3-year: 73.9%*|*57.2%	FE	FE	FE	FE
Hong 2018 [15]	Mit 8 mg/m^2^, Epi 60 mg/m^2^, and DDP 60 mg/m^2^ on day 1, 5-FU 450 mg/m^2^ and Leu 30 mg/m^2^ on day 8	3D-CRT, IMRT	DDP 30 mg/m^2^ every week	5-year: 72.0%*|*67.9%	5-year: 61.1%*|*50.0%	5-year: 76.2%*|*70.8%	5-year: 79.9%*|*70.0%	95.3%|92.5%	FE
Zhang 2019 [16]	Gem 1 g/m^2^ (day 1 and 8) and DDP 80 mg/m^2^ every 21 days for 3 cycles	IMRT	DDP 100 mg/m^2^ every 21 days for 3 cycles	3-year: 94.6%*|*90.3%	3-year: 85.3%*|*76.5%	3-year: 91.1%*|*84.4%	3-year: 91.8%*|*91.0%	97.9%*|*98.7%	75.7%*|*55.7%

epirubicin: Epi; paclitaxel: Pac; cisplatin: DDP; gemcitabine: Gem; carboplatin: CBP; docetaxel: Doc; 5-fluorouracil: 5-FU; mitomycin: Mit; leucovorin: Leu; 2D/2D-CRT: 2/3-dimensional conformal radiotherapy; *IMRT* intensity modulated radiotherapy; *OS* overall survival; *FFS* failure-free survival; *DMFS* distant metastasis-free survival; *LRFS* locoregional relapse-free survival; *ORR* objective response rate; *AE* adverse event; *FE* fail to extract

^a^ AE during concurrent chemotherapy

^b^ means statistically significant

Across the eligible studies, Zhang et al showed the highest rates of 3-year survival outcomes for patients treated with IC plus CCRT (OS: 94.6% versus 90.3% in CCRT group; FFS: 85.3% versus 76.5% in CCRT group). In Frikha’s study, the IC + CCRT group had the greatest improvements in 3-year survival rates compared with CCRT group (OS: 86.3% versus 68.9%; FFS: 73.9% versus 57.2%). In the setting of 5-year survival data, Yang et al exhibited that IC plus CCRT significantly increased the efficacy against CCRT alone (OS: 80.8% versus 76.8%, *p* = 0.04; FFS: 73.4% versus 63.1%, *p* = 0.007). However, IC followed by CCRT had similar objective response rates (ORRs) compared to CCRT (e.g. Fountzilas’ study: 83% versus 85%, *p* = 0.82; Cao’s study: 98.7% versus 99.2%, *p* > 0.05). For grade ≥ 3 adverse events, the rates in the IC + CCRT group ranged from 52.0 to 75.7%, which is significantly increased in comparison with the CCRT group (ranged from 37.0 to 55.7%).

### Overall survival (OS)

**Fig. 2:**
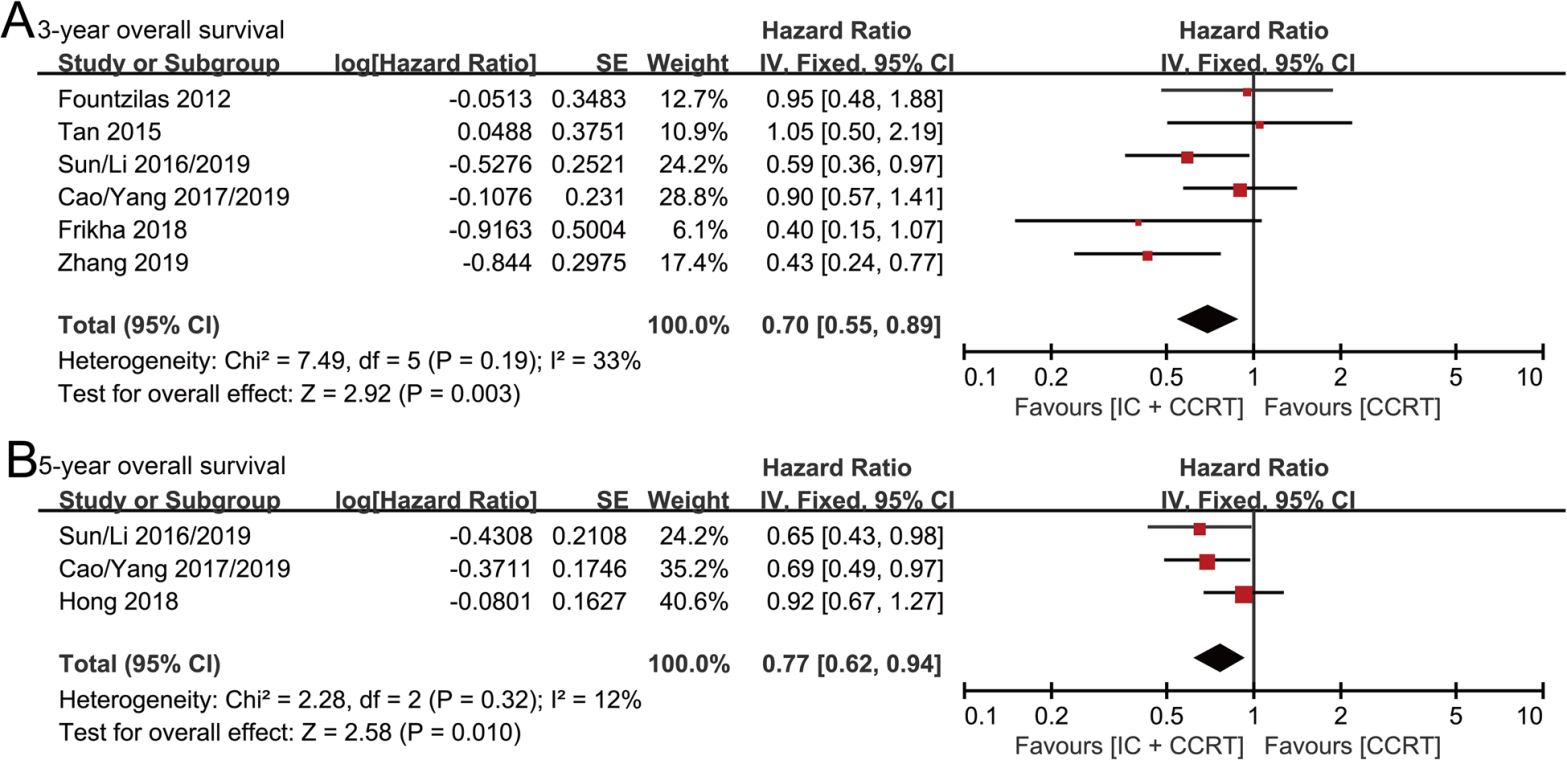
Forest plots of hazard ratios for 3-year (**a**) and 5-year (**b**) overall survival in nasopharyngeal carcinoma

3-year OS data were available from six randomized controlled trials with 1832 patients (IC + CCRT group: 921 patients; CCRT group: 911 patients). Forest plots showed patients obtained greater OS benefit from IC + CCRT compared with CCRT alone (HR: 0.70, 95% CI: 0.55–0.89, *p* = 0.003; H: *I*^2^ = 33%, *p* = 0.19) (Fig. 2a).

5-year OS data were available from three randomized controlled trials with 1435 patients (IC + CCRT group: 718 patients; CCRT group: 717 patients). Pooled results indicated that IC + CCRT led to significantly superior OS than CCRT (HR: 0.77, 95% CI: 0.62–0.94, *p* = 0.01; H: *I*^2^ = 12%, *p* = 0.32) (Fig. 2b).

### Failure-free survival (FFS)

**Fig. 3:**
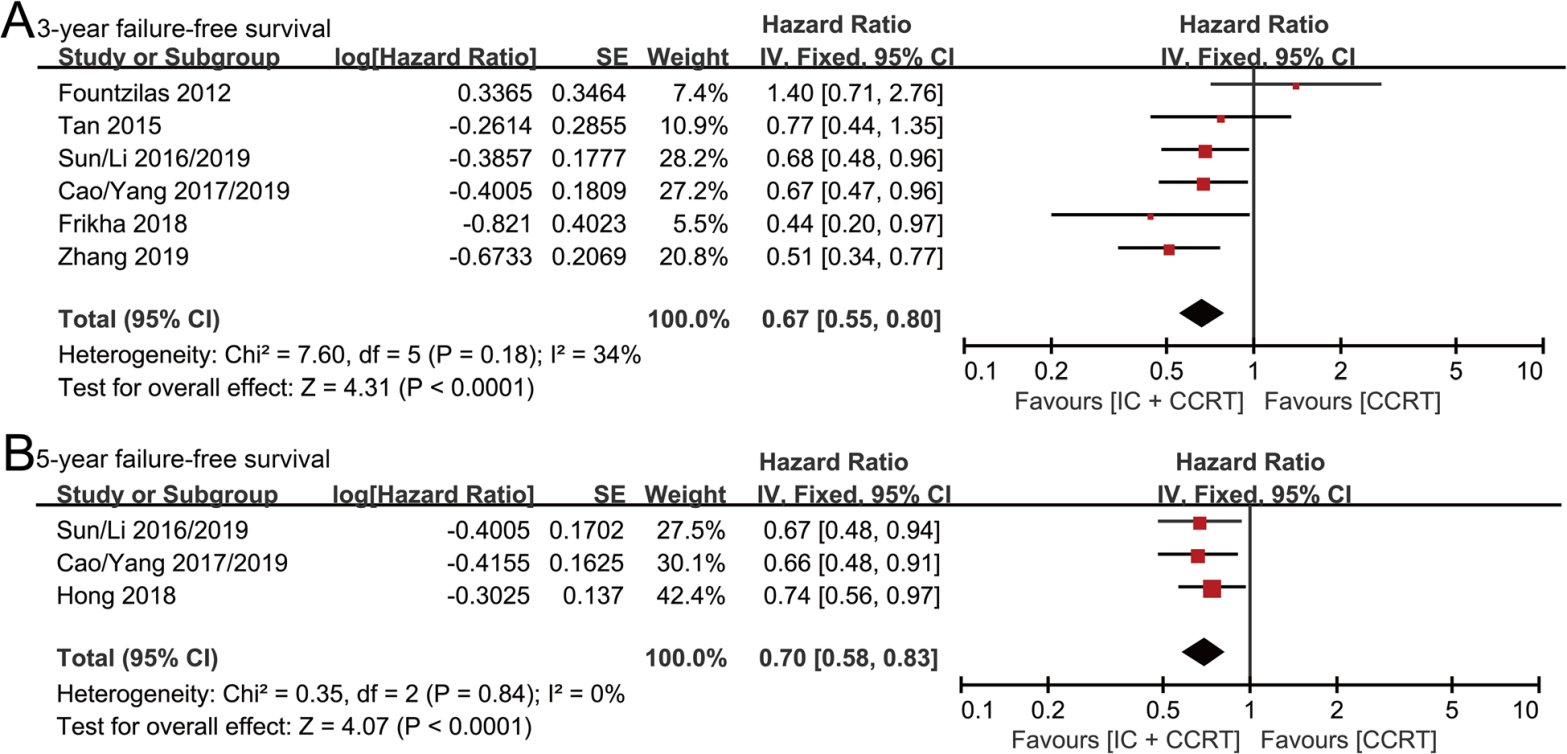
Forest plots of hazard ratios for 3-year (**a**) and 5-year (**b**) failure-free survival in nasopharyngeal carcinoma

3-year FFS data were extracted from six randomized controlled studies involving 1832 patients (IC + CCRT group: 921 patients; CCRT group: 911 patients). IC + CCRT appeared to show better FFS than CCRT (HR: 0.67, 95% CI: 0.55–0.80, *p* < 0.0001; H: *I*^2^ = 34%, *p* = 0.18) (Fig. 3a).

5-year FFS data were extracted from three randomized controlled studies involving 1435 patients (IC + CCRT group: 718 patients; CCRT group: 717 patients). IC + CCRT exhibited significant FFS superiority compared with CCRT (HR: 0.70, 95% CI: 0.58–0.83, *p* < 0.0001; H: *I*^2^ = 0%, *p* = 0.84) (Fig. 3b).

### Distant metastasis-free survival (DMFS)

**Fig. 4:**
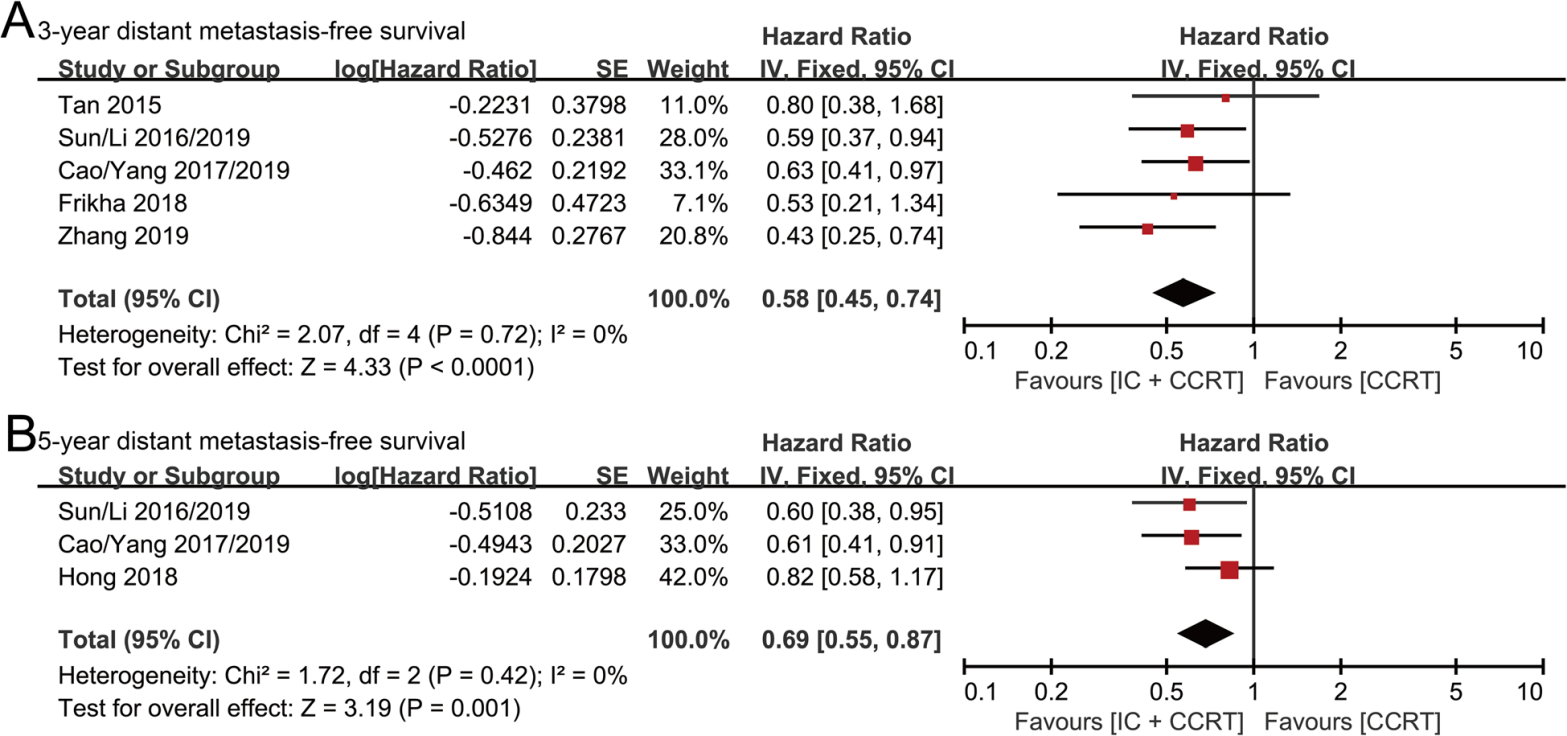
Forest plots of hazard ratios for 3-year (**a**) and 5-year (**b**) distant metastasis-free survival in nasopharyngeal carcinoma

The data of 3-year DMFS were available from five randomized controlled studies with 1691 patients (IC + CCRT group: 849 patients; CCRT group: 842 patients). The DMFS value was significantly prolonged for patients treated with IC + CCRT compared with CCRT (HR: 0.58, 95% CI: 0.45–0.74, *p* < 0.0001; H: *I*^2^ = 0%, *p* = 0.72) (Fig. 4a).

5-year DMFS data were available from three randomized controlled studies with 1435 patients (IC + CCRT group: 718 patients; CCRT group: 717 patients). A significantly lower risk of distant metastasis was shown in the IC + CCRT group versus the CCRT group (HR: 0.69, 95% CI: 0.55–0.87, *p* = 0.001; H: *I*^2^ = 0%, *p* = 0.42) (Fig. 4b).

### Locoregional relapse-free survival (LRFS)

**Fig. 5:**
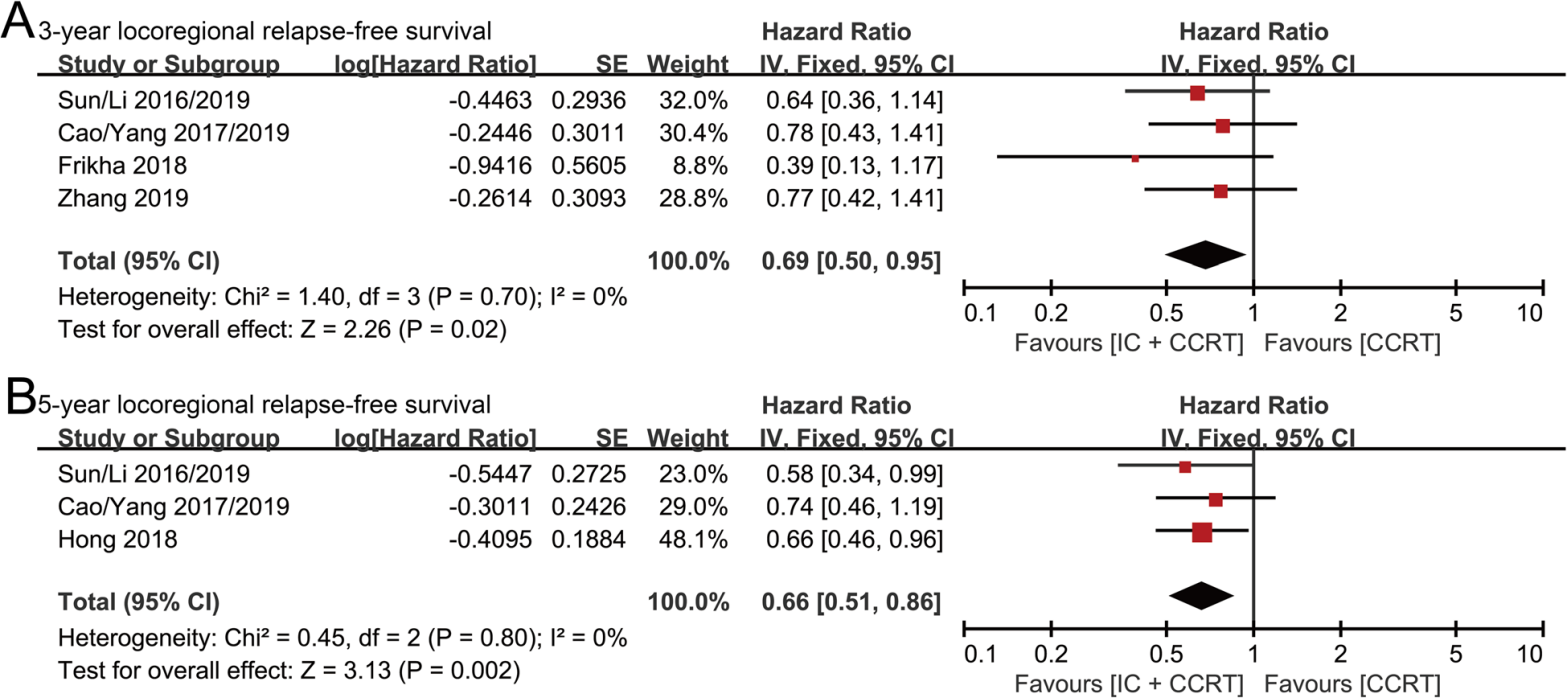
Forest plots of hazard ratios for 3-year (**a**) and 5-year (**b**) locoregional relapse-free survival in nasopharyngeal carcinoma

3-year LRFS data were collected from four randomized controlled studies involving 1519 patients (IC + CCRT group: 763 patients; CCRT group: 756 patients). Consistent with the results for DMFS, patients receiving IC + CCRT appeared to exhibit better LRFS than those receiving CCRT (HR: 0.69, 95% CI: 0.50–0.95, *p* = 0.02; H: *I*^2^ = 0%, *p* = 0.70) (Fig. 5a).

5-year LRFS data were collected from three randomized controlled studies involving 1435 patients (IC + CCRT group: 718 patients; CCRT group: 717 patients). The IC + CCRT group showed a statistically significant lower risk of locoregional relapse than the CCRT group (HR: 0.66, 95% CI: 0.51–0.86, *p* = 0.002; H: *I*^2^ = 0%, *p* = 0.80) (Fig. 5b).

### Grade ≥ 3 toxicities

**Fig. 6:**
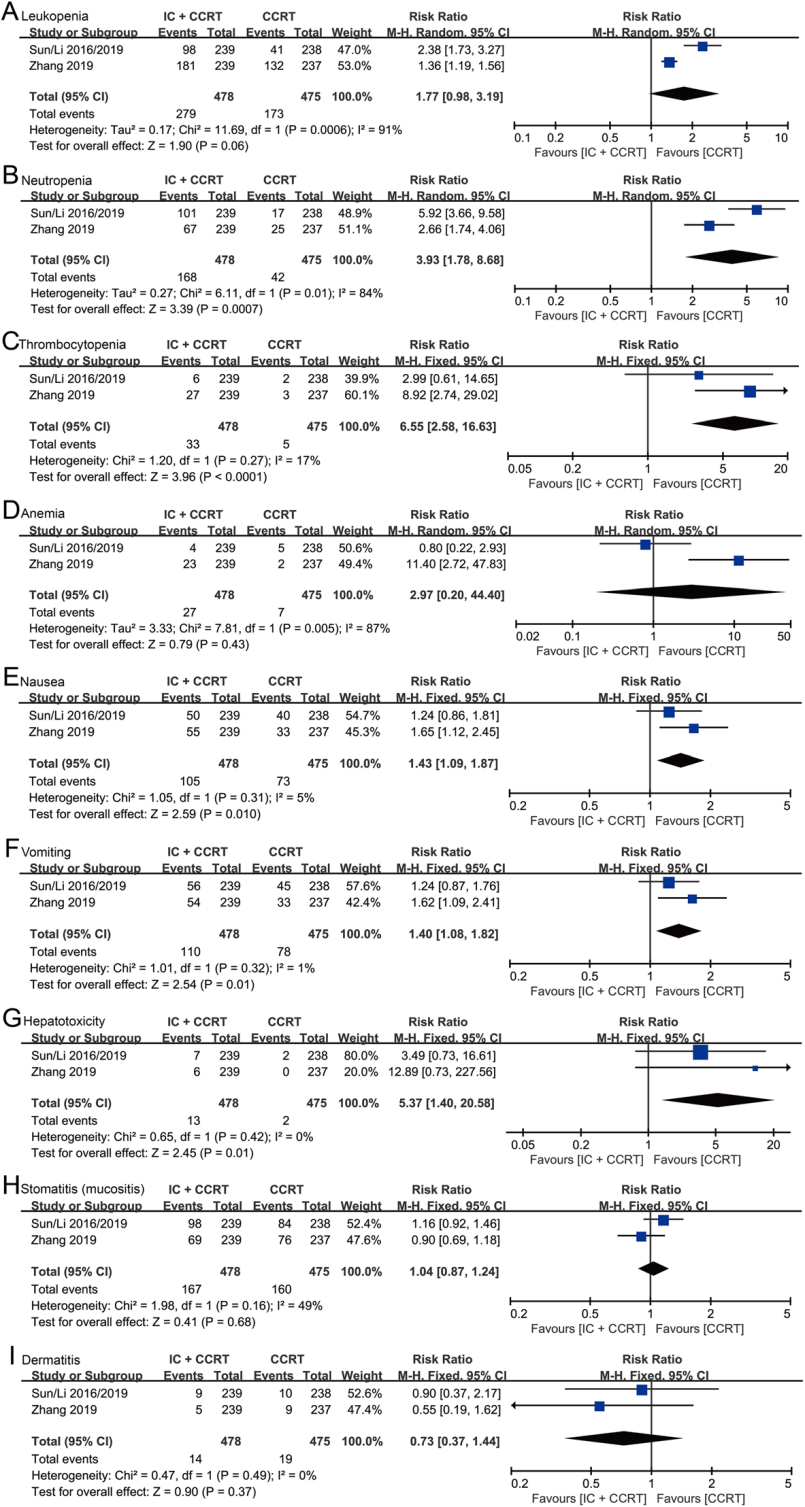
Forest plots of risk ratios for cumulative grade ≥ 3 hematological and non-hematological toxicities during overall treatment. (**a-d**) Cumulative grade ≥ 3 hematological toxicities (leukopenia (**a**), neutropenia (**b**), thrombocytopenia (**c**), and anemia (**d**)) during overall treatment. (**e-i**) cumulative grade ≥ 3 non-hematological toxicities (nausea (**e**), vomiting (**f**), hepatotoxicity (**g**), stomatitis (mucositis) (**h**), and dermatitis (**i**) during overall treatment

For grade 3 or more adverse events during the IC and CCRT, two randomized controlled trails compared the IC plus CCRT group against the CCRT group [10, 11, 16]. In hematological toxicities, there were no significant differences in leukopenia (risk ratio [RR]: 1.77, 95% CI: 0.98–3.19, *p* = 0.06) and anemia (RR: 2.97, 95% CI: 0.20–44.40, *p* = 0.43) between IC + CCRT group and CCRT group. However, the IC + CCRT group showed significantly high risks of neutropenia (RR: 3.93, 95% CI: 1.78–8.68, *p* = 0.0007) and thrombocytopenia (RR: 6.55, 95% CI: 2.58–16.63, *p* < 0.0001) than the CCRT group (Fig. 6a-d). In non-hematological toxicities, patients treated with IC + CCRT showed significantly higher risks of nausea (RR: 1.43, 95% CI: 1.09–1.87, *p* = 0.01), vomiting (RR: 1.40, 95% CI: 1.08–1.82, *p* = 0.01) and hepatotoxicity (RR: 5.37, 95% CI: 1.40–20.58, *p* = 0.01) rather than stomatitis (RR: 1.04, 95% CI: 0.87–1.24, *p* = 0.68) and dermatitis (RR: 0.73, 95% CI: 0.37–1.44, *p* = 0.37) in comparison with patients treated with CCRT (Fig. 6e-i).

**Fig. 7:**
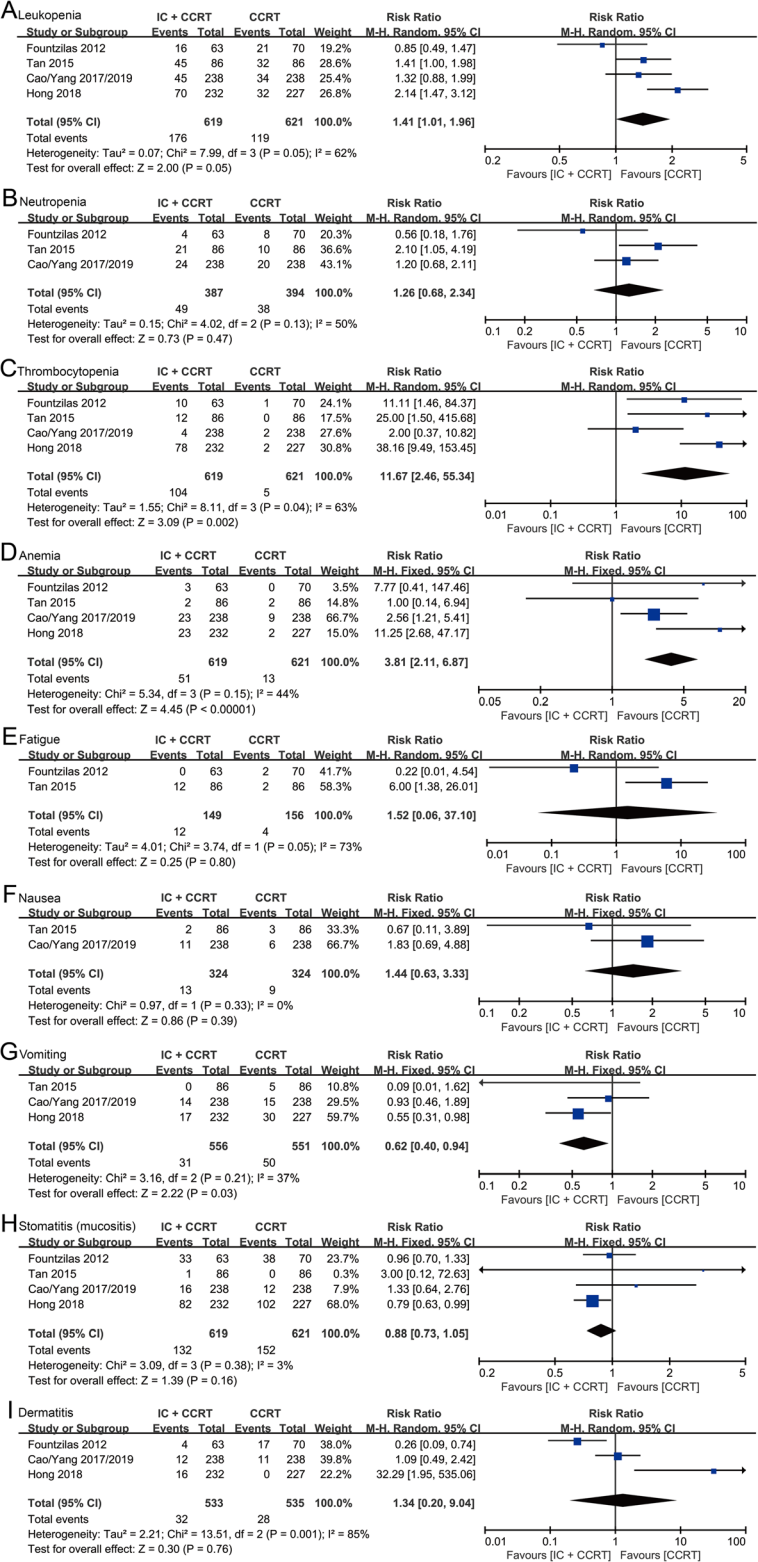
Forest plots of risk ratios for grade ≥ 3 hematological and non-hematological toxicities during concurrent chemoradiotherapy. (**a-d**) Grade ≥ 3 hematological toxicities (leukopenia (**a**), neutropenia (**b**), thrombocytopenia (**c**), and anemia (**d**)) during concurrent chemoradiotherapy. (**e-i**) Grade ≥ 3 non-hematological toxicities (fatigue (**e**), nausea (**f**), vomiting (**g**), stomatitis (mucositis) (**h**), and dermatitis (**i**) during concurrent chemoradiotherapy

For grade ≥ 3 adverse events during the CCRT, in hematological toxicities, patients in IC + CCRT group showed significantly higher risks of thrombocytopenia (RR: 11.67, 95% CI: 2.46–55.34, *p* = 0.002) and anemia (RR: 3.81, 95% CI: 2.11–6.87, *p* < 0.00001) than patients in CCRT group. There were no significant differences in leukopenia (RR: 1.41, 95% CI: 1.01–1.96, *p* = 0.05) and neutropenia (RR: 1.26, 95% CI: 0.68–2.34, *p* = 0.47) between IC + CCRT group and CCRT group (Fig. 7a-d). In non-hematological toxicities, patients treated with IC + CCRT showed a significantly higher risk of vomiting (RR: 0.62, 95% CI: 0.40–0.94, *p* = 0.03) rather than fatigue (RR: 1.52, 95% CI: 0.06–37.10, *p* = 0.80), nausea (RR: 1.44, 95% CI: 0.63–3.33, *p* = 0.39), stomatitis (mucositis) (RR: 0.88, 95% CI: 0.73–1.05, *p* = 0.16) and dermatitis (RR: 1.34, 95% CI: 0.20–9.04, *p* = 0.76) in comparison with patients treated with CCRT (Fig. 7e-i).

### Publication bias

Using the Jadad scoring scale, all enrolled trials were identified as high quality (a score of ≥3).

## Discussion

In this meta-analysis, all survival data were significantly better in NPC patients treated with IC combined with CCRT than that in patients treated with CCRT alone.

We conducted this meta-analysis to estimate the efficacy and safety of IC combined with CCRT in NPC patients. There were several early meta-analyses indicating the benefits of IC in treating patients with locoregionally advanced NPC. However, most of the studies were published before 2018 (Table 3) [17–21]. Song synthesized only four randomized clinical studies and demonstrated that IC followed CCRT performed significant treatment effects in DMFS and progression-free survival (PFS) rather than OS and LRFS [18]. In a network meta-analysis conducted by Chen, the results showed that IC plus CCRT had a higher risk of locoregional recurrence than CCRT and found no significant improvement in OS [17]. Tan analyzed six randomized controlled studies and five observation studies and displayed significant improvement in OS and PFS without the analyses of DMFS and LRFS [21]. Moreover, the inclusion of retrospective studies might increase the bias of the analysis. Although Ouyang’s pairwise meta-analysis confirmed the benefit in OS, PFS, DMFS and LRFS in NPC, patients in four of 10 included studies were treated with radiotherapy alone without concurrent chemotherapy [19]. Thus, we considered that the previous meta-analysis might not fully demonstrate the efficacy of IC + CCRT in the treatment of NPC compared with CCRT. In order to minimize the bias, we selected prospective and clinical registered randomized controlled clinical trials as the eligible studies.

**Table 3:** Summary of the cited meta-analyses and this study

Survival outcomes^a^	Total Patients	OS	FFS	DMFS	LRFS
Chen 2015 [17]	206	HR 0.70, 95% CI 0.39–1.26	FE	RR 0.51, 95% CI 0.28–0.95	RR 1.65, 95% CI 0.95–2.86
Song 2015 [18]	798	HR 0.52, 95% CI 0.21–1.29	HR 0.66, 95% CI 0.49–0.90	HR 0.60, 95% CI 0.39–0.98	HR 0.66, 95% CI 0.16–2.65
Ouyang 2019 [19]	1418	FE	FE	FE	FE
Chen 2018 [20]	1193	HR 0.75, 95% CI 0.57–0.99	HR 0.70, 95% CI 0.56–0.86	HR 0.68, 95% CI 0.51–0.90	HR 0.70, 95% CI 0.48–1.01
Tan 2018 [21]	2802	HR 0.77, 95% CI 0.60–0.98	HR 0.69, 95% CI 0.57–0.84	HR 0.63, 95% CI 0.47–0.83	HR 0.66, 95% CI 0.45–0.96
Wang	2311	3-year: HR 0.70, 95% CI 0.55–0.89; 5-year: HR 0.77, 95% CI 0.62–0.94	3-year: HR 0.67, 95% CI 0.55–0.80; 5-year: HR 0.70, 95% CI 0.58–0.83	3-year: HR 0.58, 95% CI 0.45–0.74; 5-year: HR 0.69, 95% CI 0.55–0.87	3-year: HR 0.69, 95% CI 0.50–0.95; 5-year: HR 0.66, 95% CI 0.51–0.86

*OS* overall survival; *FFS* failure-free survival; *DMFS* distant metastasis-free survival; *LRFS* locoregional relapse-free survival; *HR* hazard ratio; 95% *CI* 95% confidence interval; *FE* fail to extract

^a^ data of randomized clinical trials

Over 70% of newly diagnosed NPC patients were classified as locoregionally advanced diseases [22]. Although IMRT combined with concurrent chemotherapy improved the locoregional control, long-term survival outcomes were poor. Distant recurrence might be a major reason for the treatment failure in long-term survived patients [23–25]. The efficacy of IC in the IC + CCRT group was due to the lower incidence of distant metastatic recurrence than that in the CCRT group. In Li’s study, patients from the IC plus CCRT group showed significantly better 5-year DMFS 88% versus 79.8%; *p* = 0.030) [11], while the corresponding figures reported by Yang et al. were 82.8% versus 73.1%, *p* = 0.014 [13].

Patients could achieve better response rates and have longer survival outcomes with the administration of a more effective chemotherapeutic regimen. That is why the efficacy of IC plus CCRT in NPC is controversial [26–38]. A phase II randomized clinical study compared induction docetaxel + cisplatin plus CCRT against CCRT alone, indicating IC significantly increased 3-year OS, and positive effects on PFS and DMFS [39]. However, another phase II clinical study showed that IC of cisplatin combined with paclitaxel and epirubicin followed with CCRT did not significantly improve OS and PFS compared with CCRT alone in NPC [8]. Moreover, a randomized phase II-III study reported induction gemcitabine, carboplatin, and paclitaxel combined with CCRT had no significant differences in OS, disease-free survival (DFS) and DMFS compared with CCRT alone in patients with locoregionally advanced NPC [9]. A previously prospective clinical study proved that gemcitabine combined with cisplatin might be better than fluorouracil plus cisplatin in the first-line treatment of recurrence/metastatic NPC [40]. A retrospective study showed no significant difference in survival outcomes between induction cisplatin plus gemcitabine and cisplatin in combination with fluorouracil and docetaxel for the treatment of locoregionally advanced NPC [41]. Several ongoing clinical studies might be leading to evaluate the benefit and risk of different induction chemotherapeutic regimens in patients with locoregionally advanced NPC. For instance, NCT03604965, NCT03503136, and NCT02512315. The verification of the value of these treatment strategies is awaited.

Grade ≥ 3 adverse events were more frequent in the IC + CCRT group. During IC + CCRT, the most prominent grade ≥ 3 adverse events were neutropenia, thrombocytopenia, nausea, vomiting and hepatotoxicity. During CCRT, the most prominent grade 3 or more adverse events were thrombocytopenia, anemia and vomiting. However, these toxicities were uncomplicated, tolerated and manageable. We observed that there were no significant differences in radiotherapy related toxicities, comprising stomatitis (mucositis) and dermatitis, between the two groups. For late toxicities, Li et al. reported that the incidence of grade ≥ 3 late adverse events was 8.8% in the IC followed by CCRT group and 9.2% in the CCRT group [11]. Yang’s study also showed similar rates of late toxicities between IC + CCRT and CCRT alone group and auditory toxicities were the most common late adverse events [13].

There are several limitations in this analysis. First, different regimens and cycles of IC and CCRT might influence the survival outcomes. Second, two/three-dimensional conformal radiotherapy (2D/3D-CRT) and intensity modulated radiotherapy (IMRT) were included in the studies. Although the advent of IMRT had been demonstrated to promote a higher local tumor control rate [23], several studies had shown no significant advantage between 2D-CRT and IMRT in DMFS [24]. Third, late adverse events data were limited for further analyses. Fourth, as the EBV is an important prognostic factor, in this meta-analysis, there is no important biomarker data to suggest that which group of patients based on EBV DNA level has benefited from IC plus CCRT as compared to CCRT alone. Li′s trial is the only study in this meta-analysis that performed post-hoc subgroup analysis and demonstrated that patients with EBV ≥ 6000 copies/ml had FFS benefit when received IC followed by CCRT as compared to CCRT [11].

## Conclusion

This systematic review and meta-analysis demonstrated that, compared with CCRT alone in patients with locoregionally advanced NPC, the addition of IC to CCRT achieved favorable survival rates, and could significantly improve survival outcomes, including OS, FFS, DMFS and LRFS. As the majority of eligible studies have taken place in endemic areas, the results might not be entirely applicable to patients in non-endemic regions (e.g. EBV- patients). Additionally, it should be further explored the best selection of patient subgroups who will get the most benefit from IC plus CCRT as well as the selection of the most effective regimens for induction chemotherapy.

## Data Availability

All the published articles and data were available online.

## Abbreviations

IC: Induction chemotherapy
CCRT: Concurrent chemoradiotherapy
NPC: Nasopharyngeal carcinoma
ESMO: European Society for Medical Oncology
NCCN: National Comprehensive Cancer Network
PRISMA: Preferred Reporting Items for Systematic Reviews and Meta-analyses
OS: Overall survival
FFS: Failure-free survival
DMFS: Distant metastasis-free survival
LRFS: Locoregional relapse-free survival
PFS: Progression-free survival
DFS: Disease-free survival
HR: Hazard ratio
CI: Confidence interval
RR: Risk ratio
2D/3D-CRT: Two/three-dimensional conformal radiotherapy
IMRT: Intensity modulated radiotherapy
ORR: Objective response rate
AE: Adverse event
FE: Fail to extract
